# Musculoskeletal disorders and quality of life for Chilean teachers during the COVID-19 pandemic at the academic year-end

**DOI:** 10.3389/fpubh.2024.1277578

**Published:** 2024-05-06

**Authors:** Gustavo Vega-Fernández, Carlos Gonzalez-Torres, María Solis-Soto, Pablo A. Lizana

**Affiliations:** ^1^Laboratory of Epidemiology and Morphological Sciences, Instituto de Biología, Pontificia Universidad Católica de Valparaíso, Valparaíso, Chile; ^2^Programa de Magister en Ciencias Biológicas, Instituto de Biología, Pontificia Universidad Católica de Valparaíso, Valparaíso, Chile; ^3^Instituto de Ciencias de la Salud, Universidad de O’Higgins, Rancagua, Chile

**Keywords:** quality of life, mental health, physical health, school teachers, musculoskeletal disorders, academic period

## Abstract

**Introduction:**

Schoolteachers have reported multiple demands contributing to poor perceptions regarding their quality of life and high rates of musculoskeletal disorders. However, there are few studies about the association between musculoskeletal disorders and quality of life from the end of the academic period during the COVID-19 pandemic.

**Objective:**

Evaluate musculoskeletal disorders rates and their association with quality of life perceptions among teachers from the last academic period during the COVID-19 pandemic.

**Participants and methods:**

A total sample of 161 Chilean schoolteachers was included in a cross-sectional study musculoskeletal disorders prevalence was evaluated using the Standardized Nordic Questionnaire, and quality of life was evaluated through the Short-Form 12 Health Survey Instrument. A logistic regression was applied to evaluate the association between musculoskeletal disorders and quality of life perceptions adjusted by gender, age, and contract type.

**Results:**

98% of teachers have suffered from some type of musculoskeletal disorders during the last 12 months, and 64% have had six or more painful regions. Women showed a higher musculoskeletal disorders rate than men. The group of teachers with the most musculoskeletal disorders (≥p50) saw significantly greater risk of low scores on the physical (OR: 2.16; *p* < 0.05) and mental components (OR: 4.86; *p* < 0.01) of quality of life, regardless of gender, age, and contract type.

**Conclusion:**

High musculoskeletal disorders rates suggest that preventive and informative actions must be taken regarding these disorders in order to protect teachers’ mental and physical health, considering the effects of the school year and the COVID-19 health crisis.

## 1 Introduction

The arrival of the COVID-19 pandemic in early 2020 led to quarantines and lockdowns for billions of people worldwide (1, 2). Confinement due to COVID-19 generated structural changes for the daily activities of most people (3, 4). In this sense, one job measure adapted worldwide to avoid spreading the SARS-CoV-2 virus was changing from in-person work to remote working in a wide range of job areas (5). The occupational format of remote working combined with COVID-19 confinement has been described as one of the main causes in the rise of occupational stress among working populations in general (6), with reports associating this phenomenon with the presence of musculoskeletal pain and deteriorating quality of life (QoL) with a subsequent impact on public health (7).

Teachers saw their work format severely impacted during the pandemic due to school closures, particularly due to increased work demand, less time for planning in-person tasks and transforming them into online formats, and precarious organizational factors in work (8, 9). In this context, reports during the COVID-19 health crisis indicated that schoolteachers saw significant QoL decreases compared to before the pandemic, due to longer work hours and deteriorated work-family balance (8, 10). Remote working among teachers was also reported as a factor associated with increased burnout, stress, and high emotional demands (11, 12) together with reports of significant mental health deteriorations like higher levels of distress, worsened lifestyle quality, and increased emotional exhaustion (13–15). In this regard, pre-pandemic reports indicated high rates of musculoskeletal disorders (MSD) among schoolteachers linked with significant QoL particularly their mental QoL (16–18). The evaluation of MSDs in Chilean teachers is of great importance due to the high prevalence of these disorders and their association with a low perception of quality of life (QoL). Reports before the pandemic by COVID-19 indicated that 91% had experienced some MSD in the last 12 months, and 28.86% had had pain in 6 or more body regions. Conversely, teachers without MSDs reported higher QoL scores than those with MSDs. In addition, those teachers with the highest prevalence of MSD (≥p75) showed a significantly increased risk of having low scores on the physical (OR: 2.82) and mental (OR: 2.65) components of the QoL (16). This report suggests that preventive and informative actions regarding MSDs should be taken to protect the mental and physical health of teachers, considering the multiple risk factors to which they are exposed due to their working conditions in Chile and worldwide. Therefore, although there is a substantial body of literature on the impaired mental health of teachers (8–15), the evaluation of MSDs in teachers needs to be assessed due to their high prevalence and association with QoL (16, 17).

In this sense, due to remote working among teachers during the pandemic, there have been reports of high exposure to job stress and heavy psychosocial demands, which in turn have led to more sedentary behavior and deficient ergonomic practices, as long workdays in front of screens have led to developing various MSD (19–22). However, both at the global level and in Chile, there are no reports about the rates of MSD associated with teachers’ QoL in the context of the pandemic and especially during the end of the academic calendar, when teachers report greater time and effort spent on fulfilling the timeframes stipulated by their employers, thereby generating stress increases by up to 20% compared with the first months of the school year (23, 24). In addition, a UNESCO-conducted survey revealed that in Chile, about one-third of teachers work at two or three different schools daily, and nearly 20% have additional employment outside of teaching. These educators often perform extra work-related tasks at home or after hours (25). This perceived workload is highlighted in various studies as a potential health risk factor for Chilean teachers (8, 10, 16, 17, 26). In this regard, there is a research gap on the simultaneous impact of two critical periods that affect teachers’ QoL and MSD, such as the COVID-19 pandemic and the end of the academic year-end. The following research problem focuses on understanding how the COVID-19 pandemic has affected the prevalence of MSD and QoL among Chilean teachers at the end of the last academic year. The pandemic has significantly altered teaching practices, possibly increasing stress and changing teachers’ working conditions, which could influence the prevalence of MSD and their perception of QoL. However, the objective of the present study is to evaluate the prevalence of MSD and the QoL among Chilean school teachers at the end of the last academic school year during the COVID-19 pandemic. Therefore, we hypothesize that if we consider the new working conditions during the end of the school year, coupled with the pandemic context, and taking into account the particularly uncertain health, social, and political context of Chile (23–26), teachers will exhibit high prevalences of MSD and a deteriorated QoL, mainly in its mental component.

## 2 Materials and methods

### 2.1 Participants

166 teachers responded to the survey, of which six were excluded due to unsatisfactory responses to different instruments (exclusion criterion), leaving a total of 161 participants for analyses. Of these 161 teachers, 79.5% were female. The mean age was 40.2 years (SD ± 11.2 years) (for women 39.7 years and for men 42.5 years). Regarding marital status, 50.3% were married or lived in a steady relationship, 41% were single, and 8.7% were divorced. 62.1% had an indefinite-term contract. Concerning school types, 35.5% of the teachers belonged to public schools, 51.4% to subsidized private schools, and 13.1% to unsubsidized private schools. Finally, 58.4% of the teachers reported having children (see Table 1).

**Table 1:** Sociodemographic characteristics, prevalence of musculoskeletal disorders and quality of life according to gender and age.

Variables	Total sample	Gender			Age		
		male (*n* = 29)	female (*n* = 132)	*p*	≤44 (*n* = 109)	≥45 (*n* = 52)	*p*
Age^a^	40.236 ± 11.215	42.46 ± 12.23	39.65 ± 10.94	0.162^b^	33.550 ± 5.568	54.25 ± 5.762	<0.001
Marital status							
Single	66 (40.99)	14 (48.28)	52 (39.39)	0.569^c^	56 (51.38)	10 (19.23)	<0.001^c^
Married/Partnered	81 (50.31)	12 (41.38)	69 (52.27)		52 (47.71)	29 (55.77)	
DWW^e^	14 (8.70)	3 (10.34)	11 (8.33)		1 (0.92)	13 (25.00)	
Type of contrat							
Fixed-term	61 (37.89)	9 (31.03)	52 (39.39)	0.401^d^	49 (44.95)	12 (23.08)	<0.05^d^
Indefinite-term	100 (62.11)	20 (68.97)	80 (60.61)		60 (55.05)	40 (76.92)	
Type of school							
Public (state)	66 (40.99)	11 (37.93)	43 (32.58)	0.835^c^	37 (33.94)	17 (32.69)	0.959^c^
Private (subsidized)	87 (54.04)	15 (51.72)	72 (54.55)		59 (54.13)	28 (53.85)	
Particular (non-subsidized)	20 (12.42)	3 (10.34)	17 (12.88)		13 (11.93)	7 (13.46)	
Number of child							
Without	67 (41.61)	14 (48.28)	53 (40.15)	0.422	60 (55.05)	7 (13.46)	<0.001
With	94 (58.39)	15 (51.72)	79 (59.85)		49 (44.95)	45 (86.54)	
MSD							
<p50	58 (36.02)	14 (48.28)	44 (33.33)	0.129^d^	42 (38.53)	16 (30.77)	0.337^d^
≥p50	103 (63.98)	15 (51.72)	88 (66.67)		67 (61.47)	36 (69.23)	
PCS	45.68 ± 8.13	47.68 ± 7.72	45.24 ± 8.18	0.140^b^	45.76 ± 7.78	45.51 ± 1.23	0.964^b^
<*T*-Score	54 (33.54)	13 (44.38)	41 (31.06)	0.115^d^	32 (29.36)	22 (42.31)	0.104^d^
≥*T*-Score	107 (66.46)	16 (55.17)	91 (68.94)		77 (70.64)	30 (57.69)	
MCS	33.25 ± 10.84	36.47 ± 14.13	32.54 ± 9.90	0.191^b^	31.85 ± 1.02	36.18 ± 1.48	0.008^b^
<*T*-Score	16 (9.94)	7 (24.14)	9 (6.82)	0.005^d^	7 (6.42)	9 (17.31)	0.031^d^
≥*T*-Score	145 (90.06)	22 (75.86)	123 (93.18)		102 (93.58)	43 (82.69)	

^a^Data are expressed as mean and standard deviation.

^b^Wilcoxon Test.

^c^Fisher’s exact test.

^d^Chi-squared.

^e^Divorced Widow Widower.

MSD, Musculoskeletal Disorders; <p50, less than 6 painful regions; ≥p50, 6 or more painful regions.

PCS, Physical Component Summary; MCS, Mental Component Summary; *T*-Score, 50 indicate good QoL perception while scores below 50 indicate poor QoL perception.

*p* < 0.05.

### 2.2 Procedures

A cross-sectional study was done between the months of November and December 2021, the final month of academic activities among schoolteachers in Chile (27). Snowball sampling was used for data gathering (28) with teachers contacted via social media (Facebook and Instagram) due to the impossibility of physical contact arising from the pandemic. The main criterion for the inclusion of teachers is that they are teaching. Each professor went through the instruments online using the SurveyMonkey platform (SurveyMonkey, San Mateo, CA) with detailed instructions, research objectives, and the researchers’ contacts in case of any doubts. Before taking the surveys, the participants read and signed informed online consent on the same platform, detailing that their participation was totally anonymous, voluntary, and with a confidentiality commitment from the researchers, as well as being totally without remuneration, compensation, or economic incentives in any part of the study. This study was approved by the Ethics Committee at Pontificia Universidad Católica de Valparaíso (BIOEPUCV-H 393–2021).

### 2.3 Instruments

The descriptive variables gathered included gender, age grouped into two categories (≤44 years and ≥ 45 years) according to the cutoff points used in the Chilean National Health Survey (29), marital status (single, married/cohabitating, divorced/widowed), number of children, school-age children, type of school where they taught by funding category (public, private, charter) (30) and contract type (fixed term or indefinite). The dependent variable was QoL, and the independent variable was the presence of MSD.

QoL perceptions were evaluated with the Short-Form 12 Health Survey (SF-12), validated for the Chilean population (31). The survey consists of 12 Likert-type personal appreciation questions grouped into two summary measurements: the Physical Health Component (PHC) and the Mental Health Component (MHC). Participants’ scores for each scale and summary measurement were transformed into a 0–100 scale, followed by calculating a z-score and t-score value for PHC and MHC using internationally standardized methods (32, 33). Values above *T*-Score 50 indicated good QoL perceptions, while scores below *T*-Score 50 indicated poor QoL perceptions.

For MSD evaluation, we used the Standardized Nordic Questionnaire for Musculoskeletal Symptoms, validated for the Chilean population (34) and previously used among Chilean schoolteachers (16, 17). The survey comprises a graphic representation of a human body’s posterior view, divided into nine different anatomical regions including neck, shoulders, upper back, lower back, elbows, wrists/hands, hips/thighs, knees, ankles/feet. For each of these areas, there is a “yes” or “no” question that assesses the prevalence of self-reported musculoskeletal symptoms experienced by the respondents over the last 12 months (At any time during the last 12 months, have you had problems (pain, aches, discomfort) in the following parts of your body?). The symptoms may include aches, pain, or discomfort in these body segments. The prevalence rates were calculated based on the proportion of teachers who reported MSDs in any body part to the total number of respondents. In addition, the segments have been grouped to analyze major body segments, generating the following analysis segments: neck/shoulder, back, upper limb, and lower limb. Therefore, Standardized Nordic Questionnaire was crafted to evaluate issues related to the musculoskeletal system within a specific population, focusing on identifying the body parts where these problems are concentrated.

### 2.4 Statistical analyses

Data analyses were done using STATA v16 software for Windows (StataCorp. 2019. Stata Statistical Software: Release 16. College Station, TX: StataCorp LLC). Descriptive statistics were processed using medians with standard deviations (M ± SD) for continual variables, and frequencies with percentages for categorical variables (*n*, %). Sociodemographic variables were compared between teachers by gender. MSD rates were presented as frequency and percentage (n, %) and evaluations were done on subjects with the greatest number of painful regions grouping them into percentile 50 according to the previous 12 months (p50: ≥ 6 regions). Sociodemographic characteristics, QoL, and MSD were compared between the PHC and MHC QoL categories according to the standardized t-score (< 50 and ≥ 50 respectively). QoL was also compared in each of its components (PHC and MHC), among teachers with 6 or more painful regions (≥ p50). Median comparisons were done according to data distribution according to the Shapiro–Wilk normality test (*t*-test: parametric; Mann–Whitney: non-parametric). Chi-squared and Fisher’s Exact tests were used to analyze categorical variables. A logistic regression was also done to analyze the association between the most prevalent cases of MSD (≥ p50), with QoL in its PHC and MHC summary measures, adjusted by age, gender, and contract type. Finally, to evaluate the models’ goodness of fit, a Hosmer-Lemeshow test was used where values above 0.05 indicated that the model fit the data. The significance alpha value was 0.05.

## 3 Results

### 3.1 Sociodemographic characteristics of the study group

Table 1 presents the sociodemographic characteristics, MSD prevalence, and teachers’ QoL by gender and age category. We can observe a significant association in the gender and MHC categories for QoL (*p* < 0.01) where over 90% of women were below the *T*-Score. On the other hand, the age categories allow us to distinguish significant associations in marital status (*p* < 0.001), contract type (*p* < 0.05), number of children (*p* < 0.001) and the MHC area on QoL (*p* < 0.05) where 94% of teachers aged 44 or less fell below the *T*-Score.

### 3.2 MSD prevalence

MSD rates by age and gender appear in Table 2, indicating that the body regions with the highest MSD rates regardless of age or gender are the lower back and neck, at 83.85 and 83.23%, respectively. Along with this point, having 6 or more body regions with MSD occurred among 63.98% of respondents, while Any MSD stood at 98.14% of respondents. We can also observe that teachers over 45 years old and female teachers reported a higher rate of general MSD compared to younger teachers and male teachers.

**Table 2:** Prevalence of musculoskeletal disorders according to age and gender in Chilean teachers.

Variables	Musculoskeletal disorders				
	Total sample	Age		Gender	
		≤44	≥45	Male	Female
Neck	134 (83.23)	87 (79.82)	47 (90.38)	21 (72.41)	113 (85.61)
Shoulders	112 (69.57)	72 (66.06)	40 (76.92)	19 (65.52)	93 (70.45)
Neck/Shoulders	143 (88.82)	94 (86.24)	49 (94.23)	26 (89.66)	117 (88.64)
Elbows	70 (43.48)	41 (37.61)	29 (55.77)	9 (31.03)	61 (46.21)
Wrist/Hands	118 (73.29)	78 (71.56)	40 (76.92)	20 (68.97)	98 (74.24)
Any upper limb	155 (96.27)	104 (95.41)	51 (98.08)	28 (96.55)	127 (96.21)
Upper back	119 (73.91)	81 (74.31)	38 (73.08)	19 (65.52)	100 (75.76)
Low back	135 (83.85)	94 (86.24)	41 (78.85)	20 (68.97)	115 (87.12)
Any back	148 (91.93)	101 (92.66)	47 (90.38)	24 (82.76)	124 (93.94)
Hips/Thigs	89 (55.28)	60 (55.05)	29 (55.77)	10 (34.48)	79 (59.85)
Knees	82 (50.93)	48 (44.04)	34 (65.38)	15 (51.72)	67 (50.76)
Ankles/Feet	62 (38.51)	36 (33.03)	26 (50.00)	9 (31.03)	53 (40.15)
Any lower limb	124 (77.02)	80 (73.39)	44 (84.62)	20 (68.97)	104 (78.79)
Any MSD	158 (98.14)	107 (98.17)	51 (98.08)	28 (96.55)	130 (98.48)
≥p50	103 (63.98)	67 (61.47)	36 (69.23)	15 (51.72)	88 (66.67)
Without MSD	3 (1.86)	2 (1.83)	1 (1.92)	1 (3.45)	2 (1.52)

Data are expressed as frequency (percentage).

Musculoskeletal Disorders; ≥p50, 6 or more painful regions.

### 3.3 Association between MSD and QoL

The association between sociodemographic characteristics and MSD with teachers’ QoL appears in Table 3. The PHC on QoL has a significant association with the p50 of MSD (*p* = 0.023). On the other hand, in the MHC of QoL, there are significant associations in age, contract type, and p50 category (*p* < 0.01). Significant associations allow us to observe that most teachers below the *T*-Score for MHC are those age 44 or less and indicating that they had 6 or more body regions with MSD. Teachers with indefinite work contracts also had better mental health (87%).

**Table 3:** Sociodemographic characteristics and prevalence of musculoskeletal disorders according to physical and mental health components of quality of life.

Variables	Quality of Life					
	PCS			MCS		
	<*T*-Score	≥*T*-Score	*p*	<*T*-Score	≥*T*-Score	*p*
Age^a^	39.196 ± 10.216	42.296 ± 12.826	0.258^b^	39.448 ± 10.911	47.375 ± 11.769	0.009^b^
≤44	77 (71.96)	32 (59.26)	0.104^d^	102 (70.34)	7 (43.75)	0.031^d^
≥45	30 (28.04)	22 (40.74)		43 (29.66)	9 (56.25)	
Marital status						
Single	46 (42.99)	20 (37.04)	0.640^c^	61 (42.07)	5 (31.25)	0.660^c^
Married/partnered	53 (49.53)	28 (51.85)		72 (49.66)	9 (56.25)	
DWW^e^	8 (7.48)	6 (11.11)		12 (8.28)	2 (12.50)	
Type of contrat						
Fixed-term	41 (38.32)	20 (37.04)	0.874^d^	59 (40.69)	2 (12.50)	0.027^d^
Indefinite-term	66 (61.68)	34 (62.96)		86 (59.31)	14 (87.50)	
Type of school						
Public (state)	38 (35.51)	16 (26.93)	0.647^c^	50 (34.48)	4 (25)	0.753^c^
Private (subsidized)	55 (51.40)	32 (59.26)		77 (53.10)	10 (62.50)	
Particular (non-subsidized)	14 (13.08)	6 (11.11)		18 (12.41)	2 (12.50)	
Number of child						
Without	47 (43.93)	20 (37.04)	0.403^d^	62 (42.76)	5 (31.25)	0.375^d^
With	60 (56.07)	34 (62.96)		83 (57.24)	11 (68.75)	
School child						
Yes	38 (35.51)	19 (35.19)	0.967^d^	54 (37.24)	3 (18.75)	0.142^d^
No	69 (64.49)	35 (64.81)		91 (62.76)	13 (81.25)	
P50						
<6 painful regions	32 (29.91)	26 (48.15)	0.023^d^	47 (32.41)	11 (68.75)	0.005^d^
≥6 painful regions	75 (70.09)	28 (51.85)		98 (67.59)	5 (31.25)	

^a^Data are expressed as mean and standard deviation.

^b^Wilcoxon Test.

^c^Fisher’s exact test.

^d^Chi-squared.

^e^Divorced Widow Widower.

MSD, Musculoskeletal Disorders; p50, 50 % MSD.

PCS, Physical Component Summary; MCS, Mental Component Summary; *T*-Score, 50 indicate good QoL perception while scores below 50 indicate poor QoL perception.

*p* < 0.05.

Finally, Table 4 reports the results of the logistic regression done between the MHC and PHC areas for QoL with MSD rates and work characteristics by gender and age. This table shows that the p50 category for MSD is associated with worse scores on both the physical and mental domains (OR = 2.160; *p* = 0.029 and OR = 4.857; *p* = 0.011), while being female (OR = 3.417; *p* = 0.041) is also a risk factor for the QoL MHC. Finally, after incorporating age as a continuous variable within the model, we can observe a trend whereas teachers’ age increases, there is a proportional decrease in the odds of having worse scores in the mental health component for QoL (OR = 0.946; *p* = 0.043).

**Table 4:** Logistic regression for the association between physical and mental health components of quality of life with prevalence of musculoskeletal disorders and work characteristics adjusted for gender and age.

Variables	PCS (T-Score)		MCS (T-Score)	
	OR [95% CI]^a^	*p*	OR [95% CI]^a^	*p*
≥p50 MSD	2.160 (1.084–4.304)	0.029	4.857 (1.446–16.308)	0.011
Gender (female)	1.527 (0.656–3.555)	0.326	3.417 (1.049–11.129)	0.041
Age^b^	0.975 (0.947–1.005)	0.106	0.946 (0.897–0.998)	0.043
Type of contrat (Fixed-term)	-		0.389 (0.076–1.988)	0.257
Hosmer-Lemeshow	0.568		0.670	

^a^OR, Odds Ratios [Confidence interval].

^b^Variable ‘Age’ was included in the model as a continuous variable.

Physical Component Summary; MCS, Mental Component Summary; *T*-Score, 50 indicate good QoL perception while scores below 50 indicate poor QoL perception.

≥p50 MSD, 6 or more regions with MSD.

*p* < 0.05.

## 4 Discussion

The study evaluated the relationship between MSD and QoL in a sample of teachers in Chile during the end of the academic period during the COVID-19 pandemic. The presence of 6 or more regions with MSDs seems to be an important risk factor for poor QoL, considering its physical and mental components, independent of age, gender, and type of contract. In our study, the prevalence of MSD in any part of the body was 98.1%, and for 6 or more parts of the body was 64.0%, these results show an increase in the prevalence of musculoskeletal disorders in teachers compared to previous studies carried out both inside and outside the country (16, 17, 35, 36). For example, while the prevalence of musculoskeletal disorders in 6 or more parts of the body in our results was 64.0%, the Solis-Soto study carried out prior to the pandemic in Bolivia reported a prevalence of 47.8% and only considering 3 or more body parts (35). Other previous studies also carried out on Chilean teachers during the pandemic present lower prevalences than those reported in this study, for example, Vega-Fernandez reported only a 28.9% prevalence of MSD in 6 or more parts of the body, a much lower value compared with our results (16), which could mean a progressive increase in teachers’ musculoskeletal disorders as the pandemic progressed.

Although the prevalence of MSD in the back, neck, and upper limbs has been previously reported as the most affected, our results suggest that its prevalence has increased during the period of the COVID-19 pandemic. Compared to one study before the pandemic, including rural and urban areas in Chile, and using the same questionnaire, it shows that any upper limbs discomfort increased by 20% (from 76 to 96.3%), back by 21% (from 71 to 92%) and neck/shoulders by 22% (from 62 to 89%). At the same time, slight variation was reported in other parts of the body, such as knees, which reported an increase of 5% (from 46 to 51%), and ankles/feet by 3% (from 36 to 39%) (16). This difference could be explained due to the change in class modality from face-to-face to virtual learning. Zyznawska and Bartecka reported that the average degree of declared back pain is significantly associated with the number of hours spent at the computer (37). Possibly during the pandemic, pre-existing complaints have intensified and become more persistent. Likewise, the most common discomforts in the body are related to the type of work and common postures exercised in the virtual classes of most schools. Sitting for long hours in front of the computer, inappropriate rest breaks, increased and inappropriate use of smart devices, improper keyboard and mouse position, uncomfortable table and chair for work, and the physical and environmental conditions of the room have been related to the increased MSD in employees using a computer (19–22, 38). Additionally, before the pandemic, most teachers did not have training in ergonomics for working in front of the computer, which could cause rapid MSD due to inadequate postures.

On the other hand, women presented higher prevalences in all parts of the body. It is consistent with other studies, which generally reported a higher prevalence and severity of MSD in women, partly explained by the higher overload of domestic tasks (36). However, during the pandemic, this difference may have deepened and decreased the work–non-work balance, especially at the beginning of the health crisis (39, 40). To those mentioned above it is essential to add that, during the study, Chilean teachers were at the end of the academic period and in the first steps toward the return to face-to-face teaching, so the amount of telework may have decreased, but the traditional work and the time dedicated to it were still at the worrying levels that have been described for years (41–44), or could even be higher, due to the greater effort required for end-of-semester activities (23).

Our study also found differences in MSD by age. People over 45 years of age have reported higher prevalences of MSD in different body parts, which may have a direct relationship with the human body’s aging process (45). However, our study found a higher prevalence in the lower and upper back areas in younger people (under 45 years of age), which could be related to lifestyle changes due to the lockdown imposed during the pandemic. Restriction on social gatherings, financial problems, and housing conditions seems particularly stressful for younger adults (46). On the other hand, younger teachers could face a career shock at the beginning of their working life (47).

In consistency with previous studies in Chile (16), our results show a significant association between MSD and QoL, both for its physical and mental components. In our study, QoL scores, especially for the mental component, have been reported to be significantly lower in women and younger people. Female teachers showed higher fatigue and anxiety with using technology for education during the Covid-19 pandemic (48–50). On the other hand, work-family and domestic and professional task conflicts could influence QoL and explain the higher techno-overload, techno-invasion, techno-complexity, and role overload reported more frequently in women than men (10, 51). Younger people have also reported lower QoL scores. It could be explained by the economic impact of the pandemic, uncertainty, and loneliness, as previously reported (52). In this sense, our results found that young teachers with more than six body regions with MSD are more at risk to have worse QoL in their mental component. In this line, by incorporating Age as a continuous variable in our regression models, it can be observed that as the teacher’s age increases, there is a proportional decrease in the probability of having worse scores in the mental health domain of QoL, reinforcing the results of previous studies mentioned above on a greater impact of teleworking on younger teachers.

## 5 Limitations and strengths

Several limitations of the study can be identified. One of them refers to the cross-sectional design of the study. For this reason, it is impossible to ensure a causal association since it is impossible to guarantee the temporality criterion. It is possible that the sample needs to be representative of teachers in Chile. As we implemented an online survey, some teachers with limited internet access or use of digital tools may have yet to be able to participate in the study, possibly in rural areas or more peripheral to the city. However, the characteristics of the sample, in general, are similar to those of a previous study where randomized sampling and face-to-face surveys were carried out in various regions of Chile (16). On the other hand, the number of responses has been lower than initially expected. This may be due to non-response survey fatigue, which has been reported before and has been more significant during the pandemic due to limitations in face-to-face activities (53). To minimize response fatigue and improve the quality of the information, the implemented survey has paid particular attention to factors that have been reported to be essential for response fatigue, such as survey length, question complexity, and question type (54).

Additionally, some variables not considered in the analysis, such as socioeconomic level, hours of work per week, and teaching level, could be potential confounders in the relationship studied and could explain some of the results.

Among the strengths of the study, we highlight in the first place the simultaneous approach to the impact of the COVID-19 pandemic and the end of the academic semester on teachers’ health. This issue is relevant to developing public and labor policies adapted to the current circumstances. Second, the study highlights the identification of particularly critical groups, such as women and younger teachers, highlighting the need to design specific and targeted interventions for these subgroups to address the challenges faced by teachers effectively.

## 6 Conclusion

This study provides insight into the impact of the COVID-19 pandemic during the end of the academic year on the health of teachers in Chile, specifically focusing on the prevalence of MSDs and their association with HRQoL. Our results indicate that working conditions during the critical period for COVID-19 at the end of the academic year have contributed to the increase of work stress and MSDs among teachers, negatively affecting their physical and mental health. The prevalence of MSDs is alarmingly high, with a marked prevalence in women and younger teachers, underscoring the need to consider gender and age dynamics in policy and public health responses.

Furthermore, this study highlights the direct relationship between MSDs and decreased HRQoL, suggesting that interventions to improve working conditions and ergonomics in telework settings are crucial. Public health policies and work practices must be adjusted to address these challenges, especially in health emergencies like the COVID-19 pandemic.

Finally, the results highlight the importance of conducting additional research to understand how working conditions during critical periods (e.g., remote work) effect teachers’ health and to develop effective strategies to improve their quality of life. This study provides valuable evidence that can inform policy decisions and institutional practices to support teacher health and well-being in times of crisis and increased workload, such as the end of the academic year.

## Data availability statement

The raw data supporting the conclusions of this article will be made available by the authors, without undue reservation.

## Ethics statement

The studies involving humans were approved by Ethics Committee at Pontificia Universidad Católica de Valparaíso (BIOEPUCV-H 393-2021). The studies were conducted in accordance with the local legislation and institutional requirements. The participants provided their written informed consent to participate in this study.

## Author contributions

GV-F: Writing – review & editing, Writing – original draft, Methodology, Investigation, Funding acquisition, Formal analysis, Data curation, Conceptualization. CG-T: Writing – review & editing, Writing – original draft, Formal analysis, Data curation. MS-S: Investigation, Writing – original draft, Writing – review & editing. PL: Conceptualization, Data curation, Formal analysis, Funding acquisition, Investigation, Methodology, Project administration, Resources, Supervision, Validation, Writing – original draft, Writing – review & editing.
